# Improved diagonal queue medical image steganography using Chaos theory, LFSR, and Rabin cryptosystem

**DOI:** 10.1007/s40708-016-0057-z

**Published:** 2016-09-09

**Authors:** Mamta Jain, Anil Kumar, Rishabh Charan Choudhary

**Affiliations:** 1 Department of Computer Science and Engineering, Mody University, Lakshmangarh, Rajasthan India; 2 Department of Neuro Surgery, School of Medicine, University of Maryland, Baltimore, MD USA

**Keywords:** LSB, Chaos theory, LFSR, Improved diagonal queue, Steganography, Rabin cryptography, Brain disease cover image

## Abstract

In this article, we have proposed an improved diagonal queue medical image steganography for patient secret medical data transmission using chaotic standard map, linear feedback shift register, and Rabin cryptosystem, for improvement of previous technique (Jain and Lenka in Springer Brain Inform 3:39–51, 2016). The proposed algorithm comprises four stages, generation of pseudo-random sequences (pseudo-random sequences are generated by linear feedback shift register and standard chaotic map), permutation and XORing using pseudo-random sequences, encryption using Rabin cryptosystem, and steganography using the improved diagonal queues. Security analysis has been carried out. Performance analysis is observed using MSE, PSNR, maximum embedding capacity, as well as by histogram analysis between various Brain disease stego and cover images.

## Introduction

In this era, various medical systems are continuously migrating into the cloud and mobile environments. In the version of the telemedicine, the doctor examines medical images along with the patient data, which are transmitted from remote places, which help in receiving medical care by expediting diagnosis and immediate treatment. Security parameters such as authentication, integrity, confidentiality, and availability have to be considered for secure transmission, where Department of Health and Human Services (DHHS), USA imposed regulations for data security and privacy under the health insurance portability and accountability act (HIPAA) of 1996, USA [1–3].

Cryptographic techniques encrypt the secret records with a password and assume that only authorized parties have access to the password [4], along with the traditional encryption schemes, chaos-based techniques are used these days, because of properties such as ergodicity, mixing property, sensitivity to initial conditions, and system parameters which can be considered analogous to ideal cryptographic properties such as confusion, diffusion, balance, and avalanche properties. Hence, many chaos-based encryption systems also proposed in last few years [5, 6].

In steganography, the very existence of sensitive data by concealing the data in nondescript areas of the carrier image, such that the changes made to the image are imperceptible, and the secret information is retrieved only by authorized person [7–9]. Various steganography techniques have used the concept of pixel-value differencing for secret data insertion [10–12]. The pixel-value differencing (PVD) scheme uses the difference value between two consecutive pixels in a block to determine how many secret bits should be embedded [13, 14]. Steganography methods' performance can be measured by the three valuable specifications: security, capacity, and visual imperceptibility [15, 16].

Since the debut of this era, one of the most intelligible terms of information technology and communication is the security of medical records and patient’s personal information like unique ID, name of patient, disease information, etc.

Anderson et al. discussed some limitations in steganography methods [17]. They approached an information theoretic method using Shannon’s theory for perfect security of data. Therefore, both steganography and cryptography are used together to accomplish the security challenges [15].

Thiyagarajan et al. proposed a new steganography methodology for hiding patient information inside a medical cover image using a dynamic key produced by graph 3 coloring problem [18].

In this paper, we have proposed an improved security system by using the chaotic 2D standard map and linear feedback shift register, as an improvement in our previous report [19]. The proposed algorithm comprises three stages, viz. (i) generation of pseudo-random sequences (pseudo-random sequences are generated by linear feedback shift register and standard chaotic map); (ii) permutation and XORing using pseudo-random sequences; and (iii) steganography using the improved diagonal queues.

This novel approach can be understood by referring the following divisions. In division 2, brief description of chaos, linear feedback shift register, Rabin cryptosystem, queue, and security system of Jain et al. [19] is discussed. In division 3, the proposed method is discussed. In division 4, security analysis is performed. The performance analysis is carried out in division 5. Finally, the work is concluded in division 6.

## Brief description of Chaos, LFSR, Rabin cryptosystem, queue, and security system of Jain et al. [19]

In this section, various techniques have been discussed as follows:

## Basics of Chaos Theory and LFSR

### Chaotic sequence

It has a large circle, susceptible to initial value, and impulsiveness. The encryption with chaos is fast, so it is broadly merged with traditional encryption. The fundamental chaotic system model is given as follows [6]:

1 $$x\left( n \right) = f\left( {x\left( {n - 1} \right)} \right),$$

where the $x\left( n \right)$ is a chaotic sequence generated by the nonlinear $f\left( . \right)$, $x\left( 0 \right)$ are the initial condition values.

The 1D logistic maps whose chaotic intervals both in [0, 1] are as follows:

2 $$X_{n + 1} = X_{n} * \mu \left( {1 - X_{n} } \right),$$

where $$\mu \varepsilon [0,4]$$ and $$X\varepsilon [0,1].$$

The compound chaotic functions whose chaotic intervals both in [-1, 1] are as follows:

3 $$F\left( x \right) = \left\{ {\begin{array}{*{20}c} {8x^{4} - 8x^{2 } + 1 \ldots .., x < 0} \\ {4x^{3} - 3x \ldots .. \ldots .., x \ge 0} \\ \end{array} } \right\}$$

The chaotic standard map has a large key space compared to other maps.

4a $$X_{n + 1} = X_{n} + K* \sin Y_{n}$$

4b $$Y_{n + 1} = Y_{n} + X_{n + 1}$$

where $X_{n}$ and $Y_{n}$ are taken modulo 2π and $K$ are constant.

Now, generate the pseudo-random sequences using Eq. (4) which in turn used for generating the synthetic images and in counting the number of rounds [6].

### Linear feedback shift register

**Fig. 1:**
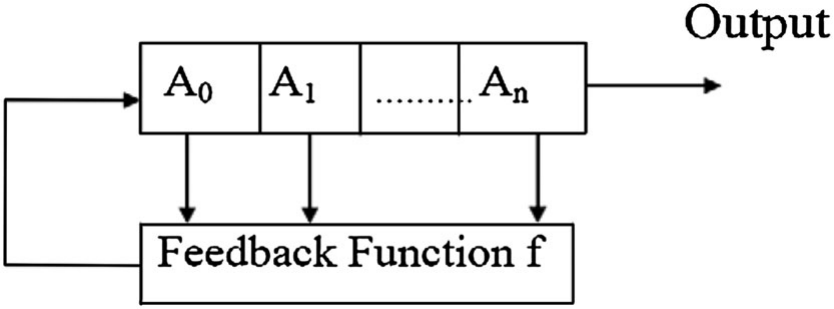
A general model of n-bit linear feedback shift register

A linear feedback shift register (LFSR) is a method for generating binary sequences [6]. Figure 1 shows a general model of an n-bit LFSR. LFSRs are extremely good pseudo-random binary sequence generators [6]. When this register is full with any given initial value (except 0 which will generate a pseudo-random binary sequence of all 0 s), it generates pseudo-random binary sequence which has very good randomness and statistical properties. The only signal necessary for the generation of the binary sequence is a clock pulse. With each clock pulse, a bit of the binary sequence is generated. An example of 4-bit LFSR is considered to demonstrate the functioning of LFSR with the feedback function $f = 1 + x + x^{4}$. Its initial bit values are used (1111). The output sequence $Z_{n}$: 011111000000001 generated by LFSR in is periodic of period 15.

Period of the sequence generated by LFSR is high if the primitive polynomial is used. To design any stream cipher system, one needs to consider the LFSR with primitive feedback polynomials as the basic building blocks. The pseudo-random sequence depends upon the initial seed value and feedback function [6].

### Rabin cryptosystem with security concern

The Rabin cryptosystem is a public key encryption method. It is established on number-theoretic problems allied to the stiffness of integer factoring and computing square roots modulo of composite number, which is straight forward when the factorization is familiar, but very composite when it is concealed. The Rabin cryptosystem requires a receiver’s public key to encrypt the text and a private key to decrypt it [4, 19].

### Queue

The linear data structure or abstractly a sequential collection is called a queue. The principal operations on the collection of data are the addition of them to the rear terminal position, known as enqueuing, and deletion of data from the front terminal position, known as dequeuing [20].

### Brief description of security system [19]

Jain et al. [19] proposed a security system which utilizes Rabin cryptosystem, diagonal queue, dynamic keys ,and Brain Disease medical cover image. The dynamic keys were considered as the secret key for the algorithm and public key for Rabin encryption. The algorithm comprises three rounds. The first round encryption using Rabin cryptosystem, the outcome is organized in various blocks and equally distributed sub-blocks (using dynamic keys). The second round, the secret cipher blocks and sub-blocks are assigned dynamically to selected diagonal queues for embedding. In third round, steganography process is performed using LSB (5th–8th bits, dynamic keys are also embedded into the cover image).

## Main scope of improvements

- Apart from secret data, metadata are also embedded into cover image, which consume lot of space of the cover image.
- Dynamic keys are inserted into the cover image. Hence, these will be easily available to the intruders, which are not desirable.
- Creation of diagonal queue is static.

## Proposed work

**Fig. 2:**
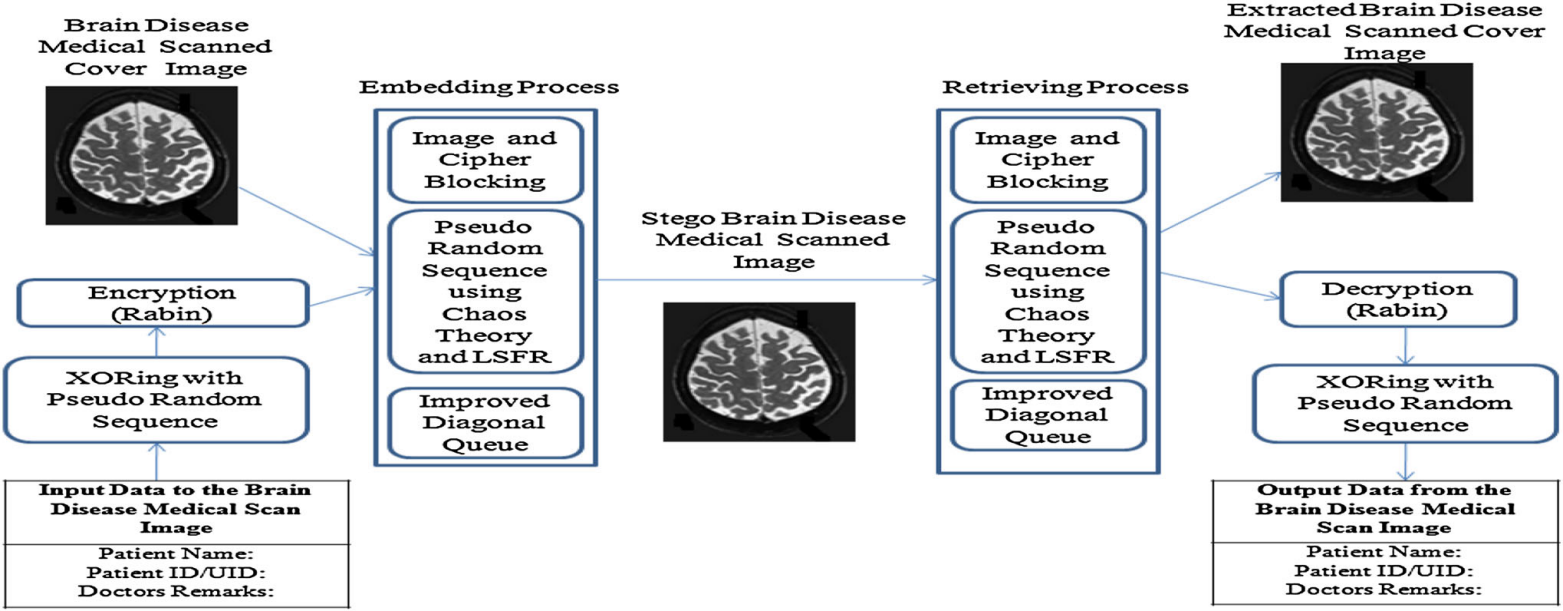
Architecture of the proposed algorithm

**Fig. 3:**
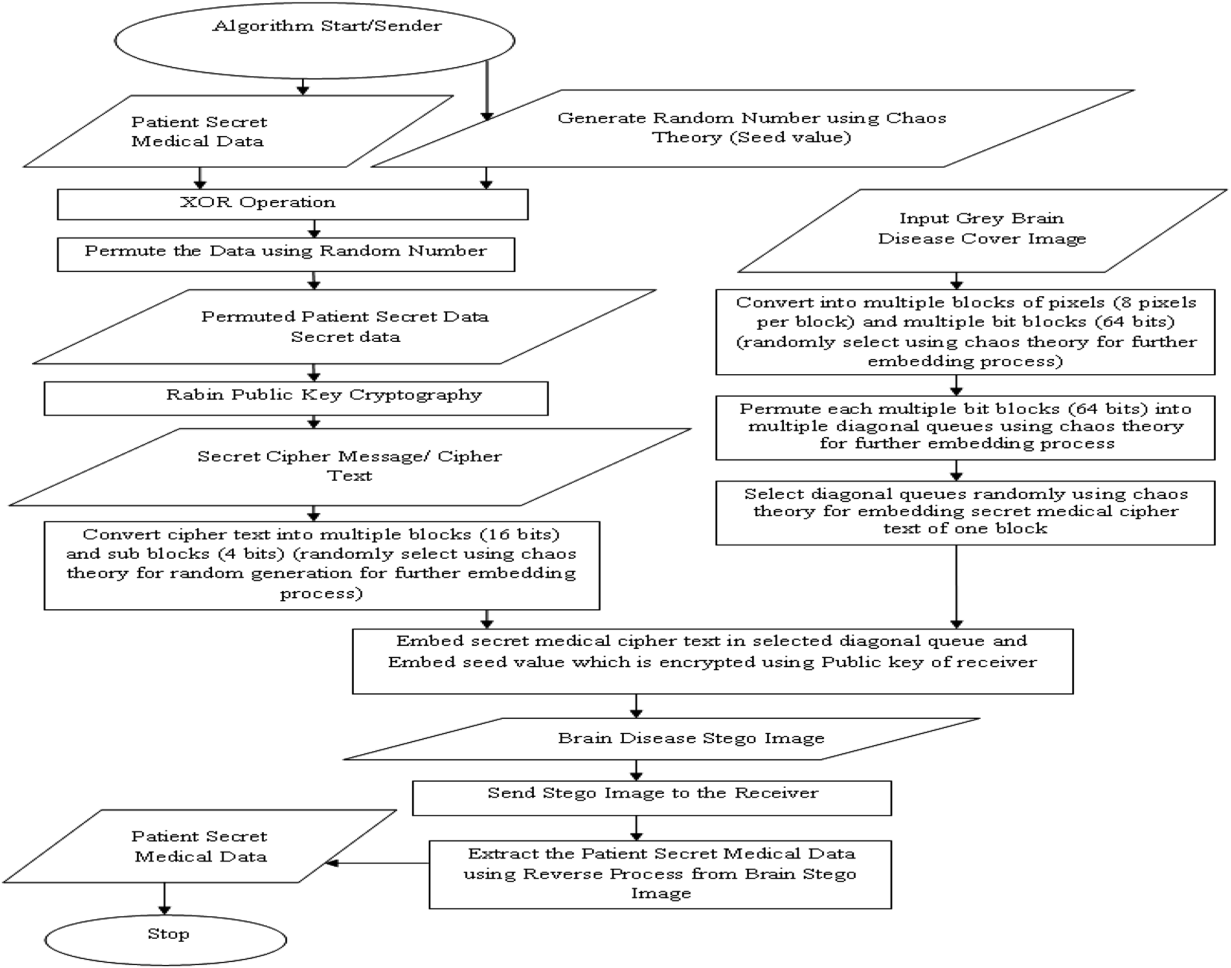
Workflow of the algorithm

In the proposed security system, it includes the input medical secret data of patient, secret keys, and grey Brain disease cover image. Figures 2 and 3 show the architecture and workflow of this algorithm.

### Cover image and secret message

In this system, select a grey Brain disease image as a cover image and a secret message of patient information, which will be embedded in the cover image.

### Chaos theory for random number generation

Here, generation of pseudo-random sequences is discussed.

- The secret key consists of three floating point numbers and one integer $(x_{0,} y_{0,} K,N)$, where $x_{0}$,$y_{0} \in (0,2\pi )$, $K$ can have any real value greater than 18.0 and $N$ is any integer value, ideally should be greater than 100.
- By iterating Eq. (4) 2 times the size of the secret data, the pseudo-random sequence is generated as $XKey$ and $YKey$
  $$For\;i = 1\; to\; 2\,*\,size\;of\;the\; \sec\; ret\; data$$
  5a $$X1\left( i \right) = \left[ {\frac{{Xkey\left( i \right)}}{{2\pi }}*256} \right];$$
  5b $$Y1\left( i \right) = \left[ {\frac{{Ykey\left( i \right)}}{{2\pi }}*256} \right];$$
  End

### Pseudo-random sequence generation using LFSR

Here, the various pseudo-random sequences are generated as follows:

- $$For i = 1 to (secret data size)/2$$.
- Generate the pseudo-random sequences $(L1(i))$ using the primitive root $, f = 1 + x^{4} + x^{5} + x^{6 } + x^{8}$, and the seed value is $X1(i)$.
- Generate the pseudo-random sequences $(L2(i))$ using the primitive root,$f = 1 + x^{4} + x^{5} + x^{6 } + x^{8}$, and the seed value is $Y1(i)$.
- Generate the pseudo-random sequences $(L3(i))$ using the primitive root,$f = 1 + x + x^{4}$, and the seed value is $(X(i) - Y1(i))(mod 15)$.
- Generate the pseudo-random sequences $(L5(i))$ using the primitive root,$f = 1 + x + x^{4}$, and the seed value is $(Y1(1)*X1(1))(mod 15)$

End

### XORING and permutation of secret data using pseudo-random sequence

- Let secret data be $S$
- Here, XOR pseudo-random sequence $(X1)$ with secret data is $S$
  6 $$S{\prime } = S( \oplus )X1$$.
- Divide the $S^{'}$ into blocks of 256 bytes.
- Permute first block elements using pseudo-random sequence $L1(1)$, second block with $L1(2),$ and so on.
- Permute first 256 blocks using pseudo-random sequence $s L2(1)$, next 256 blocks using $L2(2)$ and so on.
- Combine the entire blocks as $S^{''}$.

### Rabin cryptosystem

- Rabin encryption technique is used to encrypt the permuted secret data $S^{''}$ before embedding
  7 $$S^{'''} = RabinCryotsystem (S^{''} , Publickeyofreceiver).$$
- Now, cipher text will be divided into 16 bits blocks sequentially.
- Permute first block elements using pseudo-random sequences $L3(1)$, second block with $L3(2)$ and so on.
- After that, each block is divided into equally distributed 4 bits sub-blocks.
- Permute first sub-block elements using pseudo-random sequence $s L4(1)$, second sub-blocks with $L4(2),$ and so on.

### Diagonal queue

- Now, the cover image will be divided into a number of $8$ pixels image blocks.
- Now, organize the first image block’s $64$ bits in diagonal queues using pseudo-random sequence $s L5(1)$, second blocks using $L5(2),$ and so on.

### Diagonal queue embedding

Now, embedding the secret cipher text in the cover image using diagonal queues is done as follows:

- Encrypt seed values along with data size using public key of receiver as $F = E((Seed + Secret Data Size),Public key of Receiver)$, and embed in cover image.
- Select first 8 pixels of the cover image, and assign them random number $XKey\left( 1 \right)$ to $XKey(8)$, next $8$ pixels of the cover image, and assign them random number $XKey(9)$ to $XKey(16)$ and so on.
- One block of the cover image using pseudo-random number as discussed above point is represented by diagonal queues for embedding.
  - If the generated random value is $0$ for particular pixel, then the $8$ bits will be distributed from right to left direction.
  - If the generated random value is $1$ for particular pixel, then the $8$ bits will be distributed from left to right direction example as above.
- Embedding will be done in selected diagonal queues sequentially, using LSB technique from 5th to 8th bits using pseudo-random number $(4 + L5(i))$.

Continue this process until all the cipher data blocks are not empty and all secret cipher text is not embedded in diagonal queues sequentially and send resultant stego image to the receiver.

Example of the embedding procedure is shown in Table 1.

**Table 1:** (a–f) are cipher text blocks, cipher text sub-blocks, Brain disease cover image blocks, assignment of random number to cover image blocks, cover image block into bits and diagonal queues, respectively

Suppose we have the following data a. N blocks of permuted cipher text: 1x16 b. Permuted 4 sub-blocks of each N block: 1x4 c. M block of 8 pixels each, from cover image: 1x8 d) Now, assign pseudo-random number to each pixel for converting the above block into bits: As example the pseudo-random number generated as $01100110$ e) M block of $64$ bits each. It is obtained by converting the above block into bits as follows: If the generated random value is $0$ for particular pixel, then the $8$ bits will be distributed from right to left direction. If the generated random value is $1$ for particular pixel, then the $8$ bits will be distributed from left to right direction example as above. Using the above matrix, various diagonal queues from left to right inserted bits are created on the basis of above example. The above shown bold bits in the example are used to swap with the cipher text bits using FIFO property of queue. Now, we will select one of these eligible diagonal queues, using chaos theory of random number generation. We will also select one of the N blocks and sub-blocks using chaos theory of random number generation. We will then put the selected ciphertext bits, in selected diagonal queue at 5th to 8th bit LSB position. Example for selection of hiding position in diagonal queues using pseudo-random number is as follows: If $L5(1)$ is having value $1$ then $4 + L5\left( 1 \right)$ is $5$, i.e., secret cipher data bit will be hidden at 5th LSB position in selected diagonal. If $L5(1)$ is having value $2$ then $4 + L5\left( 1 \right)$ is $6$, i.e., secret cipher data bit will be hidden at 6th LSB position in selected diagonal. If $L5(1)$ is having value $3$ then $4 + L5\left( 1 \right)$ is $7$, i.e., secret cipher data bit will be hidden at $7$ th LSB position in selected diagonal. If $L5(1)$ is having value $4$ then $4 + L5\left( 1 \right)$ is $8$, i.e., secret cipher data bit will be hidden at 8th LSB position in selected diagonal.	

### Extraction process

Extraction of the secret medical data can be obtained as exact reverse of the encryption and embedding as discussed in above sections.

## Security analysis

Security of the proposed system is discussed as follows:

- Starting seed values are encrypted using public key of the receiver.
- As we know, slight change in seed values will generate the complete different pseudo-random sequences (using chaos).
- Seed values, for the LFSR, depend upon the pseudo-random sequence generated by the chaos.
- Hence, slight change in the starting seed value will generate different pseudo-random sequences of the LFSR.
- Permutation depends on the pseudo-random sequences.
- The complexity of the chaos is very high, and it is next to impossible to break, with existing systems.
- Also, encryption using Rabin cryptosystem increases the complexity.
- Diagonal queue generation having the complexity $2^{8}$, and permutation complexity is 15!, Hence, overall diagonal queue complexity will be $2^{8} *15!$

## Performance analysis

**Fig. 4:**
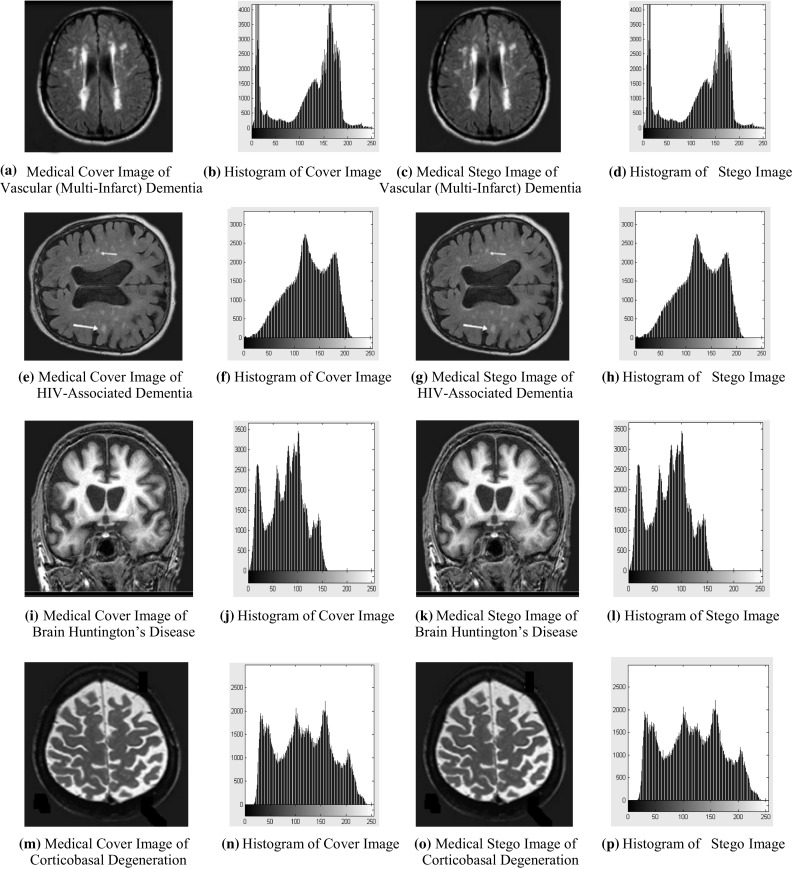
**a**, **e**, **i**, **m** are Brain disease medical cover images and **c**, **g**, **k**, **o** are their stego images, respectively, **b**, **f**, **j**, **n** are histograms of Brain disease medical cover images and **d**, **h**, **l**, **p** are their stego images histograms, respectively

The simulation and experimentation have been carried out using MATLAB. Resultant simulated outcome for different Brain disease cover images and their stego images are being displayed in Fig. 4. Histograms also show the negligible amount of difference between histogram of original Brain disease cover image and stego image. Histograms for various Brain disease cover images and their stego images are also shown in Fig. 4. The patient information used in this work is shown in Table 2.

**Table 2:** Patient secret medical data

Input data to the Brain disease medical scan cover image	Output using proposed methodology
Patient Name: XXX	Patient Name: XXX
Patient ID/UID: XX	Patient ID/UID: XX
Doctors remarks: X	Doctors remarks: X

The clause PSNR (Peak Signal-to-Noise Ratio) is a technical terminology that defines the ratio between the maximum power of a signal and the power of damaged noise. An important index to readjust the quality of reformation of steganography images is peak signal-to-noise ratio. The original cover image acts like a signal, and the noise is the defect included by some steganography mechanism. The PSNR, MSE (Mean Square Error), and maximum embedding volume values at divergent payloads for different cover images of various sizes are given in Table 3. PSNR is calculated in decibels (dB). A high-quality stego image should aspire for 40 dB and above [21].

**Table 3:** Observed Capacity, MSE, and PSNR value (different Brain disease cover images of same/different sizes with various secret cipher medical data of same/different sizes)

Brain disease cover image (*.bmp)	Brain disease cover image size (Kilo bytes)	Quantity of cipher embedded (Bytes)	Maximum embedding volume (Kilo bytes)	Percentage of embedding volume in % w.r.t (Image size)	MSE	PSNR (dB)
Vascular (Multi-Infarct) dementia	262	256	97.54	37	0.0031	76.57
Vascular (Multi-Infarct) dementia	262	1024	97.54	37	0.0062	72.17
HIV-associated dementia	262	256	93.57	36	0.0038	75.36
HIV-associated dementia	262	1024	93.57	36	0.0067	72.58
Brain Huntington’s disease	262	256	90.43	35	0.0052	75.02
Brain Huntington’s Disease	262	1024	90.43	35	0.0043	73.48
Corticobasal degeneration	262	256	88.31	34	0.0037	77.16
Corticobasal degeneration	262	1024	88.31	34	0.0057	73.27
Vascular (Multi-infarct) dementia	1048	256	408.20	39	0.0004	85.26
Vascular (Multi-Infarct) dementia	1048	1024	408.20	39	0.0025	80.39
HIV-associated dementia	1048	256	402.29	38	0.0005	84.28
HIV-associated dementia	1048	1024	402.29	38	0.0024	79.39
Brain Huntington’s disease	1048	256	397.44	37	0.0005	84.39
Brain Huntington’s disease	1048	1024	397.44	37	0.0027	79.49
Corticobasal degeneration	1048	256	385.49	36	0.0004	85.19
Corticobasal degeneration	1048	1024	385.49	36	0.0021	79.36

PSNR outcome is defined by the mean square error (MSE) for two $P*Q$ monochrome images, where $x$ as well as $y$ are image coordinates, $SG_{xy}$ (stego image) and $CV_{xy}$ (cover image), one of the images is approved a noisy surmise of the other is defined as follows:

8 $$MSE = \frac{1}{PQ}\mathop \sum \limits_{x = 1}^{P} \mathop \sum \limits_{y = 1}^{Q} (SG_{xy} - CV_{xy} )$$

9 $$PSNR = 10 \log_{10} \left( {\frac{{CV_{max}^{2} }}{MSE}} \right)$$

where $CV_{max}$ represents a maximum 255 pixel value, for 8-bit cover images [22]

Using Table 3, results are analyzed. If Brain disease cover images such as vascular (multi-infarct) dementia, HIV-associated dementia, Brain Huntington’s disease, and corticobasal degeneration of size 262 kilobytes and secret data size is 256 bytes, then PSNR and MSE values will be in the range from 75.02 to 77.16 dB and 0.0052 to 0.0037, respectively, and if data size increases to 1024 bytes then PSNR and MSE values will be in the range from 72.17 to 73.48 dB and 0.0062 to 0.0043, respectively. If cover images size increases to 1048 kilobytes and secret data size is 256 bytes, then PSNR and MSE values will be in the range from 84.28 to 85.26 dB and 0.0005 to 0.0004, respectively, and if secret data size increases to 1024 bytes then PSNR and MSE values will be 79.39–80.39 and 0.0024–0.0025, respectively.

- In vascular (multi-infarct) dementia image, maximum embedding capacity is 97.54 and 408.20 kilo bytes which is 37 and 39 %, respectively, of the image size.
- In HIV-associated dementia image, it is 93.57 and 402.29 kilo bytes, which is 36 and 38 %, respectively, of the image size.
- In Brain Huntington’s disease image, it is 90.43 and 379.44 kilo bytes, which is 35 and 37 %, respectively, of the image size.
- In corticobasal degeneration image, it is 88.31 and 385.49 kilo bytes, which is 34 and 36 %, respectively, of the image size.

So by result analysis, it can be noticed that by increasing the Brain disease cover image size and decreasing the secret data size, PSNR value will be increased up to 85.26 dB and MSE value will be decreased up to 0.0004 as well as maximum embedding capacity is increased up to 39 %. So that performance will be high with respect to PSNR, MSE, and maximum embedding capacity values.

**Fig. 5:**
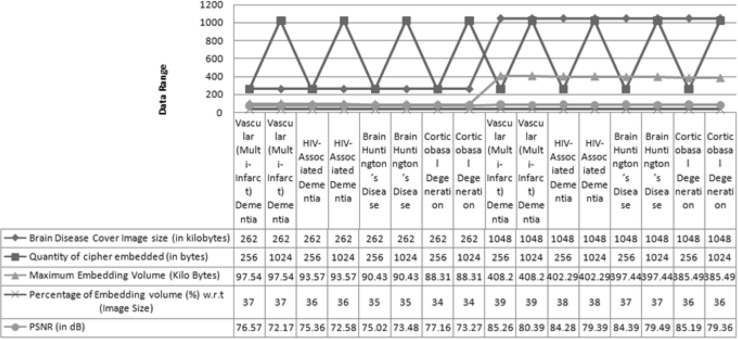
Result analysis of proposed algorithm using various performance parameters

Using Fig. 4, one can observe that there are no visual artifacts with the stego images and histograms, it is looking exactly same as corresponding original cover images. Figure 5 shows the result analysis of proposed algorithm using various performance measure parameters.

Using Table 4, results of the proposed scheme are compared with the results of the Jain et al.'s [19] work, on the basis of minimum calculated PSNR, embedding capacity, and MSE values. By comparing the results of these two articles, this article shows greater PSNR, embedding capacity, and less MSE values when simple plaintext is concealed in Brain disease cover images with same data and cover image size.

**Table 4:** Comparison with Jain et al.'s [19] work

Research article	Brain disease cover image size (Kilo Bytes)	Quantity of cipher embedded (Bytes)	Maximum embedding volume (Kilo Bytes)	Percentage of embedding volume w.r.t (Image Size)	MSE	Minimum calculated PSNR (dB)
Jain et al. [19]	262	256	84.49	32 %	0.0049	73.02
Proposed Algorithm	262	256	90.43	35 %	0.0052	75.02
Jain et al. [19]	262	1024	89.32	34 %	0.0054	70.37
Proposed algorithm	262	1024	97.54	37 %	0.0062	72.17
Jain et al. [19]	1048	256	383.49	37 %	0.0004	82.18
Proposed algorithm	1048	256	402.29	38 %	0.0005	84.28
Jain et al. [19]	1048	1024	383.49	37 %	0.0011	77.29
Proposed algorithm	1048	1024	385.49	36 %	0.0021	79.36

Using Table 5, results of the proposed scheme are compared with the results of the other works, on the basis of minimum calculated PSNR, embedding capacity, and MSE values. By comparing the results of these articles, proposed work shows greater PSNR, embedding capacity, and less MSE values when simple plaintext is concealed in cover images with same data and cover image size.

**Table 5:** Comparison with other researchers

Research article	Minimum calculated PSNR(dB)	Capacity	Visual imperceptibility
Thiyagarajan and Aghila [18]	65.53	Good	Better
Wang et al. [13]	44.20	Medium	Good
Kumar et al. [14]	44.15	Medium	Good
Wu et al. [10]	37.90	Very Low	Average
Zhang et al. [11]	36.00	Very low	Average
Chang et al. [12]	33.53	Very low	Average
Nag et al. [8]	30.48	Very low	Not good
Proposed algorithm	72.17	Very good	Best

## Conclusion and future scope

In this article, an improved secret medical data transmission scheme is proposed using the notion of opacity with respect to an improved diagonal queue least significant bits substitution using pseudo-random sequences. The secret message blocks and sub-blocks are allocated randomly using pseudo-random sequences by the sender to the Brain disease cover image blocks with respect to improved diagonal queues, which increases security levels and gives randomness to proposed algorithm. The proposed algorithm used multilevel encryption at cryptography level to provide encryption of secret patient medical data in remote places communication. At steganography level, least significant bits substitutions using chaos theory and improved diagonal queues are used to protect data. By security analysis, it is found that complexity of the encryption is very high to break, from existing system.

Proposed algorithm hides only secret medical data, embedding of metadata in Brain disease cover image is not required, so that lot of space are available in the cover image. By pseudo-random numbers using chaos theory and LSFR, no clue will be available to the intruders. By using proposed algorithm, creation of diagonal queue is dynamic. As we are hiding less data comparatively to previous technique [19] hence, from result analysis, it is concluded that PSNR, MSE values, and percentage of maximum embedding capacity are better as compared to previous work [19] and others. By histogram analysis, it is concluded that imperceptibility distortion cannot be measured from the corresponding stego images. Here, proposed technique is used for medical application and spatial domain image steganography. In future, this technique can be used for various applications using multimedia secret data, where secrecy of data is a very big challenge. We also would like to use this technique to develop some better steganography algorithms in various image steganography domains.
